# Studies of Conformational Changes of Tubulin Induced by Interaction with Kinesin Using Atomistic Molecular Dynamics Simulations

**DOI:** 10.3390/ijms22136709

**Published:** 2021-06-23

**Authors:** Xiao-Xuan Shi, Peng-Ye Wang, Hong Chen, Ping Xie

**Affiliations:** 1 School of Material Science and Engineering, Central South University of Forestry and Technology, Changsha 410004, China; sxxzszcs@163.com (X.-X.S.); hchen2017@163.com (H.C.); 2 Key Laboratory of Soft Matter Physics, Institute of Physics, Chinese Academy of Sciences, Beijing 100190, China; pywang@aphy.iphy.ac.cn

**Keywords:** kinesin, conformation of microtubule, binding energy, molecular dynamics simulation

## Abstract

The transition between strong and weak interactions of the kinesin head with the microtubule, which is regulated by the change of the nucleotide state of the head, is indispensable for the processive motion of the kinesin molecular motor on the microtubule. Here, using all-atom molecular dynamics simulations, the interactions between the kinesin head and tubulin are studied on the basis of the available high-resolution structural data. We found that the strong interaction can induce rapid large conformational changes of the tubulin, whereas the weak interaction cannot. Furthermore, we found that the large conformational changes of the tubulin have a significant effect on the interaction of the tubulin with the head in the weak-microtubule-binding ADP state. The calculated binding energy of the ADP-bound head to the tubulin with the large conformational changes is only about half that of the tubulin without the conformational changes.

## 1. Introduction

Kinesin (concretely kinesin-1) is an ATP-dependent homodimeric molecular motor that can walk progressively on microtubules (MTs), performing functions of intracellular transport [1,2,3,4]. The underlying mechanism of how the dimer walks on MTs by making use of the chemical energy of ATP hydrolysis is an interesting and challenging topic in the field of the kinesin motors [5,6,7,8,9]. To understand the mechanism, one must understand the nucleotide-dependent interactions between the kinesin motor domain (also called the head) and the MT. To that end, diverse experimental techniques have been employed [10,11,12,13,14]. It was determined that a kinesin head in nucleotide-free (*ϕ*), ATP and ADP.Pi states has a strong interaction with MTs, while in the ADP state, it has a weak interaction with MTs [10,11,12,13,14]. The strong interaction means a low equilibrium dissociation constant or a high unbinding force of the head from MTs, while the weak interaction means a high equilibrium dissociation constant or a low unbinding force. The interactions between the kinesin head and an α/β-tubulin heterodimer were also studied structurally using X-ray crystallography and cryo-electron microscopy (cryo-EM) [15,16,17]. With the high-resolution structural data and using all-atom molecular dynamics (MD) simulations, the interactions of the kinesin head in weak and strong MT-binding states with an α/β-tubulin heterodimer were also studied in detail [18,19,20].

Using relatively low-resolution cryo-EM, Morikawa et al. [17] determined that when complexed with the kinesin head in the *ϕ* state, the α/β-tubulin dimer has large conformational changes (pdb: 3J6H of 8.1 Å resolution [17]) relative to the unperturbed α/β-tubulin (pdb: 3J7I of 8.9 Å resolution [17]). With these structural data, in our previous work [21] we studied the interaction between the kinesin head and α/β-tubulin using all-atom MD simulations. It was shown that the weak affinity of the ADP-head to the α/β-tubulin with the large conformational changes (3J6H) is about 13 *k*_B_*T* smaller than that of the unperturbed α/β-tubulin (3J7I) [21].

However, as it is noted, a very important issue in these previous studies is that the all-atom MD simulations were performed with the structural data of low resolutions of 8.9 Å (3J7I) and 8.1 Å (3J6H) [17]. Moreover, the large conformational changes of the α/β-tubulin induced by the strong interaction with the kinesin head have been only observed with the low-resolution cryo-EM [17,22,23,24,25]. Whether the strong interaction between the kinesin head and α/β-tubulin can also induce the large conformational changes of the α/β-tubulin is still undetermined from the high-resolution structural studies. In this work, to address the above issues, with available high-resolution (of about 3 Å) structural data for both the kinesin head and unperturbed α/β-tubulin [16,26,27], we studied the effect of the interaction between the kinesin head and α/β-tubulin on the conformation of the α/β-tubulin using all-atom MD simulations. The simulated results showed that the strong interaction between the kinesin head and α/β-tubulin can also induce the large conformational changes of the α/β-tubulin, as the previous studies with the low-resolution structural data showed [17,22,23,24,25]. Interestingly, the large conformational changes can occur very rapidly (in the order of 10 ns). Furthermore, we studied the effect of the large conformational changes of the α/β-tubulin on the weak affinity of the tubulin to the kinesin head in the ADP state.

## 2. Results

### 2.1. The Interaction of Kinesin -Head with α/β-Tubulin Induces Rapid Large Conformational Changes of the α/β-Tubulin

To see if the strong interaction between the kinesin head and α/β-tubulin can induce the large conformational changes of the tubulin, we constructed the complex of a human kinesin *ϕ*-head (pdb: 4LNU of 2.19 Å resolution [16]) bound with an unperturbed α/β-tubulin (pdb: 1JFF of 3.50 Å resolution [27]) (see Methods), where the α/β-tubulin has no conformational change. The constructed complex is called the APO-1 system (Table 1).

**Figure 1:**
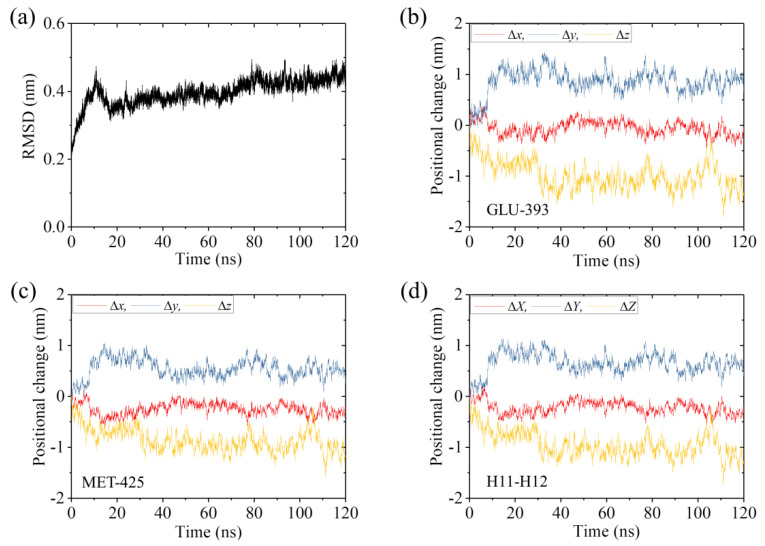
Equilibrium MD simulation results for the APO-1 system. (**a**) Temporal evolution of RMSD value. (**b**,**c**) Temporal evolution of positional changes (Δ*x*, Δ*y* and Δ*z*) of Cα atoms of GLU-393 in H11 and MET-425 in H12 of β-subunit relative to the corresponding ones at *t* = 0. (**d**) Temporal evolution of the average positional changes (Δ*X*, Δ*Y* and Δ*Z*) of Cα atoms of all residues in H11 and H12 of the β-subunit.

**Figure 2:**
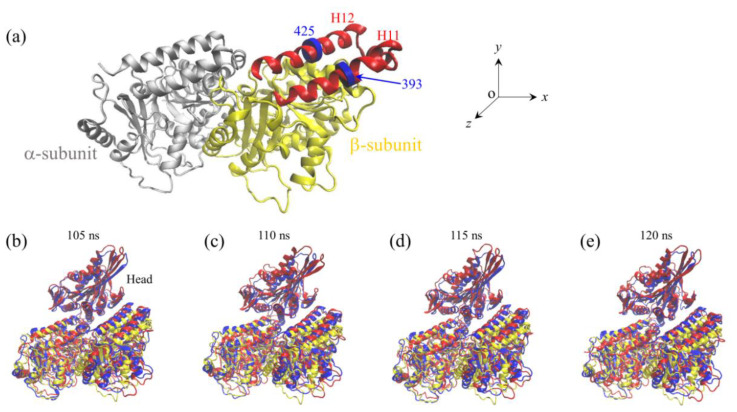
Structure of the kinesin head and α/β-tubulin. (**a**) The structure of unperturbed α/β-tubulin (drawn from pdb: 1JFF [27]). H11 helix (red) and H12 helix (red) in the β-subunit, and residue GLU-393 (blue disc) in H11 and residue MET-425 (blue disc) in H12 of the β-subunit are highlighted. (**b**–**e**) Structures of APO-1 and APO-2 systems at different simulation times. The structures of the APO-1 system at different simulation times after attaining steady state are drawn in red color, the structure of the α/β-tubulin at initial simulation time (*t* = 0) is drawn in yellow color, and the structures of the APO-2 system at different simulation times after attaining steady state are drawn in blue color.

We performed MD simulations of APO-1 system for a time of 120 ns. Two simulation results for the temporal evolution of the root mean square deviation (RMSD) for Cα atoms relative to the initial ones are shown in Figure 1a and Supplementary Materials Figure S1a (see Supplementary Materials). It is seen that RMSD increases with the time and attains the steady value in about 40 ns in Figure 1 and in about 80 ns in Supplementary Materials Figure S1, implying that the system reaches its steady state rapidly (in the order of 10 ns). To see if the large conformational changes of the α/β-tubulin can occur, in Figure 1b,c we show the temporal evolution of the changes in positions of Cα atoms of residues GLU-393 and MET-425 in β-subunit’s helices H11 and H12, respectively, where Δ*x*, Δ*y* and Δ*z* correspond to the positional changes along the *x*, *y* and *z* directions (defined in Figure 2a), respectively. As will be seen below, the two helices H11 and H12 that are located in the interface with the kinesin head have relatively large displacements. Figure 1d shows the temporal evolution of the average changes in positions of Cα atoms of all residues in β-subunit’s helices H11 and H12. The average positional changes are calculated with $\Delta X=\sum_{i}\left(\Delta x_{i}/N\right)$, $\Delta Y=\sum_{i}\left(\Delta y_{i}/N\right)$ and $\Delta Z=\sum_{i}\left(\Delta z_{i}/N\right)$, where Δ*x*, Δ*y* and Δ*z* are the changes in the position of Cα atoms of the *i*th residue in β-subunit’s helices H11 and H12 along the *x*, *y* and *z* directions, respectively, and *N* is the number of all residues in β-subunit’s helices H11 and H12. Some structures of the APO-1 system obtained at different simulation times are shown in Figure 2b–e (red), where the initial structure of the α/β-tubulin (at *t* = 0) (yellow) is also shown for comparison. From Figure 1 and Figure 2, it is evidently seen that the α/β-tubulin and in particular the β-subunit in the final steady state has large conformational changes relative to the initially unperturbed one (see also Table 2 for values of Δ*X*, Δ*Y* and Δ*Z* in the finally steady state). The large conformational changes occur rapidly and in about 40 ns the tubulin attains its final steady conformation (Figure 1). The results shown in Figure 1b–d and the structures shown in Figure 2b–e (red) correspond to those shown in Figure 1a. The results for the temporal evolution of the positional changes of the residues in β-subunit’s helices H11 and H12, which correspond to those shown in Supplementary Materials Figure S1a, are shown in Figure S1b–d (see Supplementary Information). The results shown in Supplementary Materials Figure S1 are similar to those shown in Figure 1, as expected.

**Figure 3:**
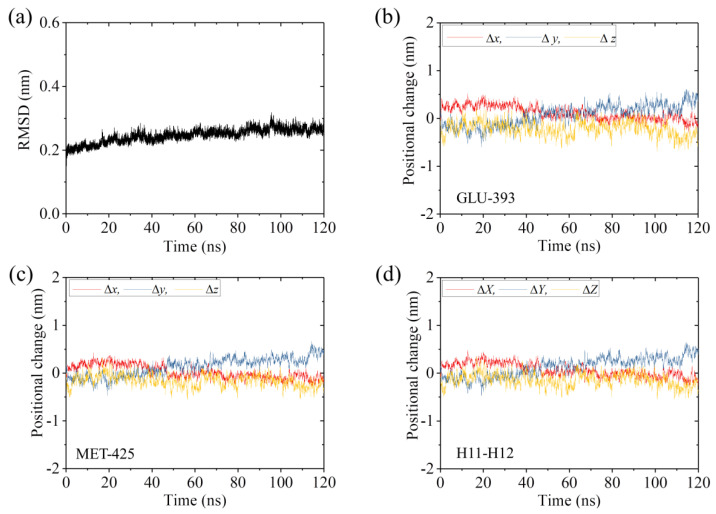
Equilibrium MD simulation results for the APO-2 system. (**a**) Temporal evolution of the RMSD value. (**b**,**c**) Temporal evolution of positional changes (Δ*x*, Δ*y* and Δ*z*) of Cα atoms of GLU-393 in H11 and MET-425 in H12 of the β-subunit relative to the corresponding ones at *t* = 0. (**d**) Temporal evolution of the average positional changes (Δ*X*, Δ*Y* and Δ*Z*) of Cα atoms of all residues in H11 and H12 of the β-subunit.

It is interesting to compare our simulated structure for the APO-1 system with that determined from X-ray crystallography. For this purpose, we performed MD simulations of the complex of the human kinesin *ϕ*-head with α/β-tubulin determined crystallographically (pdb: 4LNU of 2.19 Å resolution [16]). The complex is called the APO-2 system (Table 1). The simulation results for the temporal evolution of RMSD for Cα atoms relative to the initial ones for the APO-2 system are shown in Figure 3a. It is seen that the system attains steady state very rapidly. Similar to those shown in Figure 1b–d for the APO-1 system, in Figure 3b–d we show the temporal evolution of the changes in positions of the residues in β-subunit’s helices H11 and H12 for the APO-2 system. By comparing Figure 1b–d with Figure 3b–d, it is seen that the positional changes of the residues for the APO-2 system are much smaller than those for the APO-1 system (see also Table 2), indicating nearly no positional change of the residues relative to the initial ones for the APO-2 system, as it is expected. Some structures of the APO-2 system that are obtained at different simulation times are also shown in Figure 2b–e (blue). From Figure 2b–e it is seen that the simulated final steady structures of the APO-1 system are nearly identical to those of the APO-2 system. Since the structures of the APO-2 system at different simulation times shown in Figure 2b–e are obtained from the initial structure that is determined crystallographically, our results thus indicate that in the crystallographically determined structure (pdb: 4LNU [16]), the α/β-tubulin also has large conformational changes relative to the unperturbed tubulin. More interestingly, the conformational changes of the α/β-tubulin obtained from MD simulations are in good agreement with those determined crystallographically.

Taken together, in this section we show that the strong interaction of a kinesin head with an α/β-tubulin can rapidly induce large conformational changes of the tubulin, with a timescale in the order of 10 ns.

### 2.2. The Interaction of Kinesin ADP-Head with α/β-Tubulin Induces no Conformational Change of the α/β-Tubulin

As shown in the above section, the strong interaction between a *ϕ*-head and an α/β-tubulin can rapidly induce (in the order of 10 ns) large conformational changes of the tubulin. To see if the weak interaction between an ADP-head and an α/β-tubulin can also induce the conformational changes of the tubulin, we constructed the complex of a human kinesin ADP-head (pdb: 1BG2 of 1.80 Å resolution [26]) bound with an unperturbed α/β-tubulin (pdb: 1JFF of 3.50 Å resolution [27]) (see Methods), where the α/β-tubulin has no conformational change. The constructed complex is called the ADP-2 system (Table 1).

**Figure 4:**
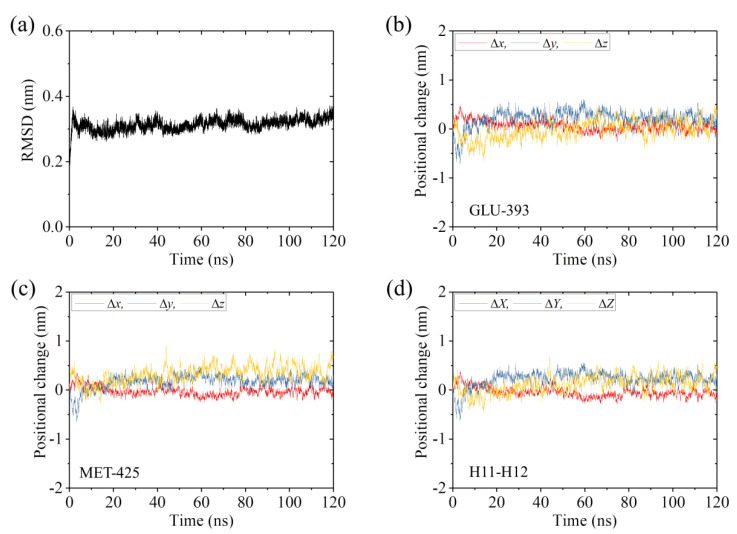
Equilibrium MD simulation results for the ADP-2 system. (**a**) Temporal evolution of the RMSD value. (**b**,**c**) Temporal evolution of positional changes (Δ*x*, Δ*y* and Δ*z*) of Cα atoms of GLU-393 in H11 and MET-425 in H12 of the β-subunit relative to the corresponding ones at *t* = 0. (**d**) Temporal evolution of the average positional changes (Δ*X*, Δ*Y* and Δ*Z*) of Cα atoms of all residues in H11 and H12 of the β-subunit.

As done for the systems studied in the above section, we also performed MD simulations of the ADP-2 system for a time of 120 ns. In Figure 4a and Supplementary Materials Figure S2a, we show two simulation results for the temporal evolution of RMSD for Cα atoms relative to the starting ones for the ADP-2 system. It is seen that the system attains steady state very rapidly. Similar to those shown in Figure 1b–d and Supplementary Materials Figure S1b–d for the APO-1 system, in Figure 4b–d and Supplementary Materials Figure S2b–d we show the temporal evolution of the changes in positions of the residues in β-subunit’s helices H11 and H12 for the ADP-2 system. By comparing Figure 1b–d and Supplementary Materials Figure S1b–d with Figure 4b–d and Supplementary Materials Figure S2b–d, it is seen that the positional changes of the residues for the ADP-2 system are much smaller than those for the APO-1 system (see also Table 2).

**Figure 5:**
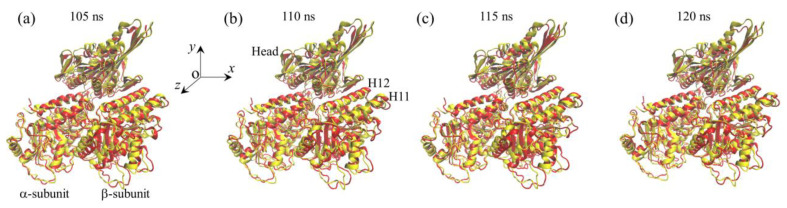
Structures of the ADP-2 system at different simulation times. The structures at different simulation times after attaining steady state are drawn in red color and the structure at initial simulation time (*t* = 0) is drawn in yellow color. (**a**) At simulation time *t* = 105 ns. (**b**) At simulation time *t* = 110 ns. (**c**) At simulation time *t* = 115 ns. (**d**) At simulation time *t* = 120 ns.

To see whether the very small positional changes of the residues and the fluctuations in the positional changes for the ADP-2 system arise from those induced by the weak interaction between the head and α/β-tubulin or from those induced by the thermal noise alone, we performed MD simulations of the isolated α/β-tubulin (Table 1). Similar to those shown in Figure 4b–d and Supplementary Materials Figure S2b–d for the ADP-2 system, in Supplementary Materials Figure S3a–d we show the temporal evolution of RMSD for Cα atoms relative to the initial ones and the temporal evolution of the changes in positions of the residues in β-subunit’s helices H11 and H12 for the isolated α/β-tubulin. Comparing Figure 4b–d and Supplementary materials Figure S2b–d with Figure S3b–d, we interestingly see that both the positional changes and fluctuations in Figure 4b–d and Supplementary Materials Figure S2b–d have similar magnitudes to those in Supplementary Materials Figure S3b–d (see also Table 2). These results thus imply that the weak interaction between the ADP-head and α/β-tubulin in the ADP-2 system induces little positional change of the residues. The fluctuations in the positional changes for ADP-2 system arise from the fluctuations in the α/β-tubulin itself. Some structures of the ADP-2 system that are obtained at different simulation times are shown in Figure 5 (red), where the initial structure of the ADP-2 system (at *t* = 0) (yellow) is also shown for comparison. From Figure 5 it is seen that the simulated steady structures of the ADP-2 system are nearly identical to the initial structure. Therefore, it is concluded that the weak interaction between the ADP-head and unperturbed α/β-tubulin induces nearly no conformational change in the α/β-tubulin.

**Figure 6:**
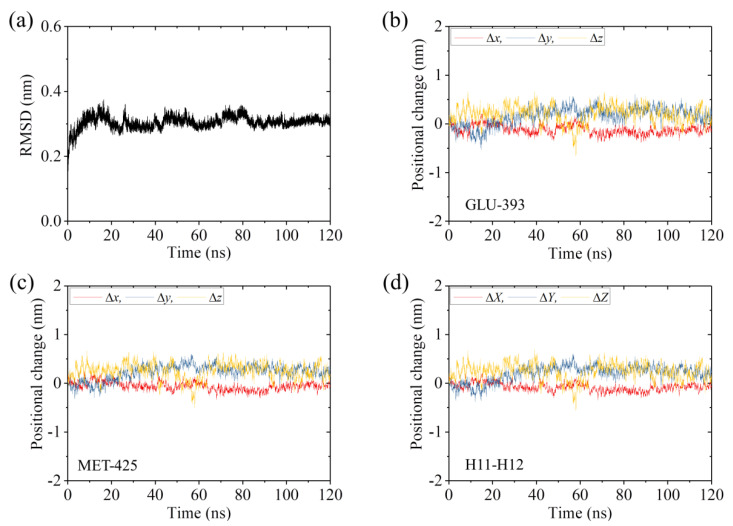
Equilibrium MD simulation results for ADP-1 system. (**a**) Temporal evolution of the RMSD value. (**b**,**c**) Temporal evolution of positional changes (Δ*x*, Δ*y* and Δ*z*) of Cα atoms of GLU-393 in H11 and MET-425 in H12 of β-subunit relative to the corresponding ones at *t* = 0. (**d**) Temporal evolution of the average positional changes (Δ*X*, Δ*Y* and Δ*Z*) of Cα atoms of all residues in H11 and H12 of the β-subunit.

**Figure 7:**
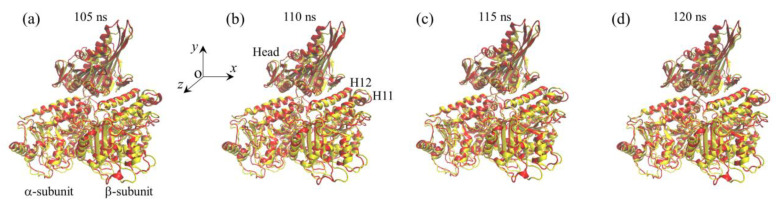
Structures of the ADP-1 system at different simulation times. The structures at different simulation times after attaining steady state are drawn in red color and the structure at initial simulation time (*t* = 0) is drawn in yellow color. (**a**) At simulation time *t* = 105 ns. (**b**) At simulation time *t* = 110 ns. (**c**) At simulation time *t* = 115 ns. (**d**) At simulation time *t* = 120 ns.

We then studied the effect of the interaction of the ADP-head with the α/β-tubulin having the large conformational changes on the structure of the α/β-tubulin. Thus, we constructed the complex of the human kinesin ADP-head (pdb: 1BG2 [26]) bound with the α/β-tubulin, where the tubulin is obtained from the MD simulations of Figure 1 at 120 ns. The constructed complex is called the ADP-1 system (Table 1). The MD simulations of the ADP-1 system were also performed for a time of 120 ns. Two simulation results for the temporal evolution of RMSD for Cα atoms relative to the starting ones for the ADP-1 system are shown in Figure 6a and Supplementary Materials Figure S4a. It is seen that the system attains the steady state very rapidly. Corresponding to that shown in Figure 4b–d and Supplementary Materials Figure S2b–d for the ADP-2 system, in Figure 6b–d and Supplementary Materials Figure S4b–d, we show the temporal evolution of the changes in positions of the residues in β-subunit’s helices H11 and H12 for the ADP-1 system. By comparing Figure 6b–d and Supplementary Materials Figure S4b–d with Figure 4b–d and Supplementary Materials Figures S2b–d, it is seen that both the positional changes of the residues and the fluctuations in the positional changes for the ADP-1 system have nearly the same magnitude as those for the ADP-2 system (see also Table 2). Thus, the weak interaction between the ADP-head and the α/β-tubulin in the ADP-1 system also induces little positional change of the residues. The fluctuations in the positional changes for the ADP-1 system also arise from the fluctuations for the α/β-tubulin itself. In addition, the simulated final steady structures of the ADP-1 system obtained at different simulation times (the red ones in Figure 7) are nearly identical to the initial structure (the yellow one in Figure 7). Therefore, we conclude that the weak interaction between the ADP-head and the α/β-tubulin with the large conformational changes also has nearly no effect on the conformation of the α/β-tubulin.

Taken together, in this section we show that the weak interaction of a kinesin head in the ADP state with an α/β-tubulin induces nearly no conformational change of the tubulin, which is in sharp contrast to the strong interaction of the *ϕ*-head with an α/β-tubulin.

### 2.3. The ADP-Head Has a Much Weaker Affinity to α/β-Tubulin with Large Conformational Changes Than That with No Conformational Change

To see how the large conformational changes of an α/β-tubulin induced by the strong interaction with a kinesin head affect the weak interaction of the ADP-head with the α/β-tubulin, we calculated the binding energy between them. We employed center-of-mass (COM) pulling and umbrella sampling (see Methods) to calculate the binding energy of the ADP-head to the α/β-tubulin with the large conformational changes and to the α/β-tubulin without the conformational changes. The former binding energy is denoted by *E*_w1_ and the latter one is denoted by *E*_w2_.

**Figure 8:**
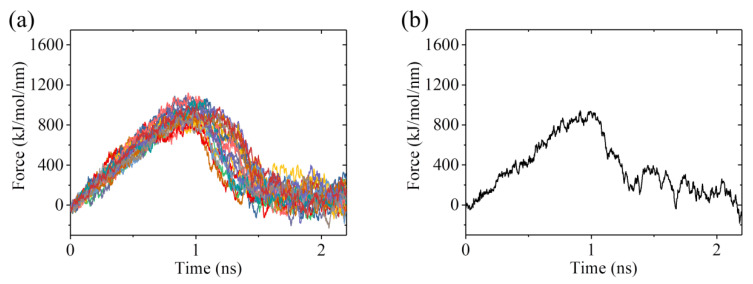
Force–time curves of pulling the kinesin head away from the α/β-tubulin for the ADP-1 system. (**a**) Twenty-two force–time curves. (**b**) The median force–time curve for the 22 curves shown in (**a**).

**Figure 9:**
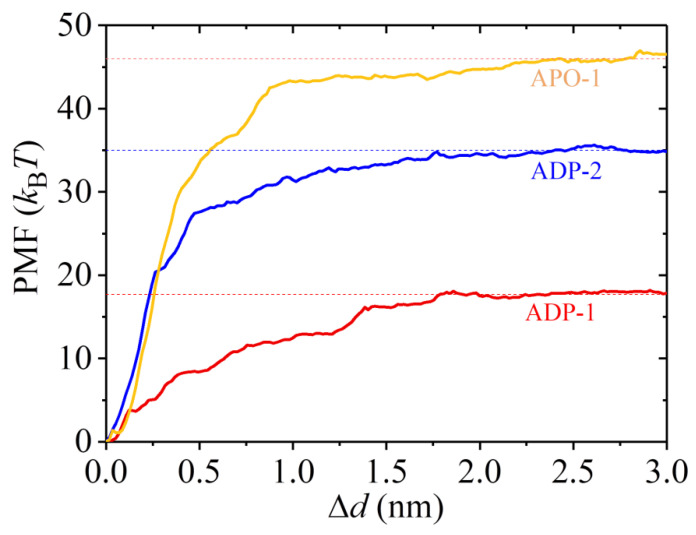
Results for the change in PMF versus the change in the distance (Δd) between COM position of the kinesin head and that of the α/β-tubulin.

To calculate *E*_w1_, we used the structures of the ADP-1 system obtained from the MD simulations shown in Figure 6 and Supplementary Materials Figure S4 after 100 ns, when the steady state of the system was attained, as the starting structures used in the pulling simulations. In Figure 8a, we show 22 curves of the pulling force versus time during the process of pulling the ADP-head away from the α/β-tubulin. It is noted that the two MD simulation results shown in Figure 6 and Supplementary Materials Figure S4 give similar pulling-time curves, and provided that the system has reached its steady state, the MD simulation results at different moments also give similar pulling-time curves. Of the 22 pulling-time curves shown in Figure 8a, we chose the median one, as shown in Figure 8b. Then, with the median pulling-time curve shown in Figure 8b, we calculated the change of the potential of mean force (PMF) as the function of the change in the distance (Δ*d*) between COM position of the head and that of the α/β-tubulin using umbrella sampling simulations. The convergent results of the change of PMF versus Δ*d* are shown in Figure 9. As anticipated, PMF increases with the increase of Δ*d* and attains the maximum steady value at Δ*d* equal to about 1.8 nm. The maximum change of PMF is about 17.7 *k*_B_*T*, leading to the binding energy of the head to the tubulin, *E*_w1_ = 17.7 *k*_B_*T*.

**Figure 10:**
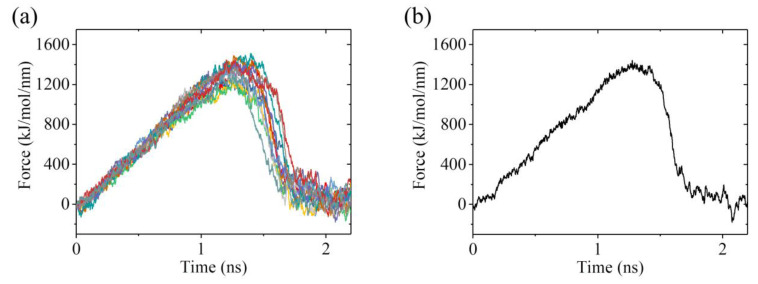
Force–time curves of pulling the kinesin head away from the α/β-tubulin for ADP-2 system. (**a**) Twelve force–time curves. (**b**) The median force–time curve for the 12 curves shown in (**a**).

To calculate *E*_w2_, we used the structures of the ADP-2 system obtained from the MD simulations shown in Figure 4 and Supplementary Materials Figure S2 after 100 ns, when the system had reached its steady state, as the starting ones used in the pulling simulations. Figure 10a shows 12 curves of the pulling force versus time during the process of pulling the ADP-head away from the α/β-tubulin. Of the 12 pulling-time curves, we chose the median one, as shown in Figure 10b. Then, with the median pulling-time curve shown in Figure 10b, we calculated the change of PMF as the function of the change in the distance (Δ*d*) between COM position of the head and that of the α/β-tubulin by using umbrella sampling simulations. The convergent results of the change of PMF versus Δ*d* are shown in Figure 9. Similar to the case for the ADP-1 system, the PMF also attained the maximum steady value at Δ*d* ≈ 1.8 nm for the ADP-2 system. The maximum change of PMF is about 35 *k*_B_*T*, indicating *E*_w2_ = 35 *k*_B_*T*, which is 17.3 *k*_B_*T* (or about 2-fold) larger than *E*_w1_ = 17.7 *k*_B_*T*.

**Figure 11:**
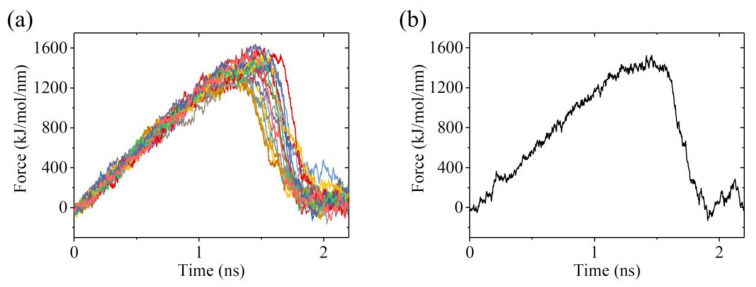
Force–time curves of pulling the kinesin head away from the α/β-tubulin for APO-1 system. (**a**) Seventeen force–time curves. (**b**) The median force–time curve for the 17 curves shown in (**a**).

For comparison, we also calculated the binding energy of *ϕ*-head to the α/β-tubulin for the APO-1 system, with the binding energy being denoted by *E*_S_. To calculate *E*_S_, we used the structures of the APO-1 system obtained from the MD simulations shown in Figure 1 and Supplementary Materials Figure S1 after 100 ns as the starting ones used in the pulling simulations. Figure 11a shows 17 curves of the pulling force versus time during the process of pulling the *ϕ*-head away from the α/β-tubulin. The median of the 17 pulling-time curves is shown in Figure 11b. Then, with the median pulling-time curve shown in Figure 11b, the change of PMF versus the change in the distance (Δ*d*) between COM position of the head and that of the α/β-tubulin was calculated, with the convergent results shown in Figure 9. From Figure 9, it is seen that the maximum change of PMF is about 46 *k*_B_*T*, indicating *E*_S_ = 46 *k*_B_*T*, which is 11 *k*_B_*T* larger than *E*_w2_ = 35 *k*_B_*T*. This is consistent with the prior experimental data showing that the *ϕ*-head has a larger binding energy to MT than the ADP-head [10,11,12,13,14].

Taken together, in this section we show that the *ϕ*-head has a high binding energy of about *E*_S_ = 46 *k*_B_*T* to an α/β-tubulin, the ADP-head has a weak binding energy of about *E*_w2_ = 35 *k*_B_*T* to an unperturbed α/β-tubulin, and the ADP-head has the weakest binding energy of about *E*_w1_ = 17.7 *k*_B_*T* to an α/β-tubulin with the large conformational changes. The weak binding energy *E*_w2_ is about 11 *k*_B_*T* smaller than the strong binding energy *E*_S_, and the weakest binding energy *E*_w1_ is about 17.3 *k*_B_*T* smaller than *E*_w2_.

## 3. Discussion

Although the low-resolution cryo-EM data indicated that the strong interaction of the kinesin head with the MT can induce large conformational changes of the local α/β-tubulin [17,22,23,24,25], this conclusion still needs to be verified by high-resolution structural studies. For this purpose, here we used the available high-resolution structural data for both the kinesin head in strong MT-binding (*ϕ*) and weak MT-binding (ADP) states [16,26] and the normally unperturbed α/β-tubulin [27] to study the interaction between the head and α/β-tubulin by using all-atom MD simulations. Our simulations showed that the strong interaction can induce large conformational changes of the α/β-tubulin, whereas the weak interaction cannot. The large conformational changes occur very rapidly (in the order of 10 ns). Furthermore, we found that the binding energy of the ADP-head to theα/β-tubulin with the large conformational changes is about 17.3 *k*_B_*T* smaller than or (only about half of) that of the normally unperturbed α/β-tubulin. It is noted that using all-atom MD simulations and with the available high-resolution structural data for both the kinesin head and α/β-tubulin, the interactions between the head and α/β-tubulin were also studied and analyzed by other researchers [18,19,20]. In those MD simulations, only the α/β-tubulin with the large conformational changes [15,16] was used. Thus, only the interaction of the head in the strong MT-binding state with α/β-tubulin and that of the head in ADP state with the α/β-tubulin with the large conformational changes were studied [18,19,20]. However, the interaction of the head in the ADP state with the α/β-tubulin without the large conformational changes was not included [18,19,20].

As is known, following the kinesin head transiting from the conformation that has a strong interaction with the α/β-tubulin to that of the weak interaction, the changed conformation of the α/β-tubulin will return elastically to the normal unchanged one. Thus, it is expected that after Pi release from the head, a short time period *t*_r_ is present when the α/β-tubulin keeps its changed conformation. Consequently, our simulation results can lead to the following deduction. Upon the kinesin head releasing Pi, for a short time period *t*_r_, the ADP-head has a binding energy to the local α/β-tubulin (*E*_w1_) that is much smaller than that to the other unperturbed α/β-tubulins (*E*_w2_). The smaller *E*_w1_ makes the ADP-head detach easily from the previous α/β-tubulin, while the larger affinity *E*_w2_ makes the ADP-head bind easily to the next α/β-tubulin. In time *t*_r_, the binding energy of the ADP-head to the local α/β-tubulin changes from *E*_w1_ to *E*_w2_. As shown elsewhere, based on the above deduction, the proposed Brownian dynamics model for the chemomechanical coupling of kinesin dimers can explain well various experimental data on dynamics of different families of N-terminal kinesin motors such as kinesin-1, kinesin-3, kinesin-5, kinesin-8, and orphan kinesin PAKRP2 [28,29,30,31,32,33]. In particular, to fit the available single molecule data on load dependences of velocity, dissociation rate, run length, etc., for the kinesin motors, using the proposed Brownian dynamics model, it was estimated that *E*_w1_ ≤ 25 *k*_B_*T* and *E*_w2_ ≥ 35 *k*_B_*T* [30,33,34,35,36,37,38], which are consistent with the values of *E*_w1_ = 17.7 *k*_B_*T* and *E*_w2_ = 35 *k*_B_*T* calculated here using all-atom MD simulations.

## 4. Methods and Materials

### 4.1. System Setup

The structure of the *ϕ*-head used in our MD simulations for the APO-1 system is based on the structural data, pdb: 4LNU (2.19 Å) [16] (Table 1). The structure of the head in the ADP state used in our MD simulations for the ADP-1 system and ADP-2 system is based on the structural data, pdb: 1BG2 (1.80 Å) [26] (Table 1). The structure of α/β-tubulin with no conformational changes used in our MD simulations for the APO-1 system, ADP-2 system and the isolated α/β-tubulin is based on the structural data, pdb: 1JFF (3.50 Å) [27] (Table 1), where a GTP molecule is bound to the α-subunit and a GDP molecule is bound to the β-subunit. The structure of α/β-tubulin with the large conformational changes used in our MD simulations for the ADP-1 system is based on the structural data obtained from our MD simulations of the APO-1 system at the steady state, i.e., the structural data at the simulation time of 120 ns (Table 1). The structure of the *ϕ*-head complexed with α/β-tubulin used in our MD simulations for the APO-2 system is based on the structural data, pdb: 4LNU (2.19 Å) [16] (Table 1), where the designed ankyrin repeat protein (DARPin) is removed. The missing atoms in the structures involved are added by using the software Swiss-PdbViewer [39].

The systems of the kinesin head complexed with the α/β-tubulin are built up via three steps, as was done in our previous work [21]. First, we performed molecular replacements with CHIMERA [40] using the structural data, pdb: 4LNU (2.19 Å) [16], as the starting model. Second, we performed the energy minimizations twice. Third, the systems were equilibrated for 100 ps at 300 K and 1 bar pressure in the NVT ensemble and NPT ensemble, respectively. For the ADP-head, the topology files of the ADP molecule were generated by the online PRODRG server.

### 4.2. MD Simulations

Here, we used the similar MD simulation procedures to those we used before [21,41]. The simulation conditions were the same as those described before [21,41]. We used GROMACS4.6 [42] with AMBER99SB force field [43] to do MD simulations. We used TIP3P water potential [44]. Solvent and necessary ions were added with favorable concentrations. Counter-ions were added to neutralize the system. We ran the MD simulations at 300 K and 1 bar. The time step was set as 2 fs and the output data were updated every 5 steps. All chemical bonds were constrained using the LINCS algorithm [45]. The short-range electrostatics interaction and the cutoff for van der Waals interaction was set as 1 nm. The Particle Mesh Ewald (PME) algorithm [46] was used for calculations of long-range electrostatics. Velocity-rescaling temperature coupling [47] and Berendsen pressure coupling [48] were used. The energy minimization was performed for 5 × 10^4^ steps using the steepest descent method.

During equilibrium MD simulations of the system of the kinesin head complexed with α/β-tubulin, we restricted residues 224 through 243 of a β-sheet in the α-subunit. These restricted residues are far away from the interface between the head and α/β-tubulin. The simulation box has a dimension of 13 × 12 × 10 nm^3^, with 13 nm, 12 nm and 10 nm in the *x*, *y* and *z* directions (see Figure 2a), respectively. During equilibrium MD simulations of the isolated α/β-tubulin, we also restricted residues 224 through 243 and the simulation box has a dimension of 13 × 9 × 9 nm^3^, with 13 nm, 9 nm and 9 nm in the *x*, *y* and *z* directions, respectively.

We used umbrella sampling simulations [49] to calculate the change in PMF of pulling the kinesin head away from the α/β-tubulin. To obtain the initial configurational windows, we restricted residues 224 through 239 of the β-sheet in α- and β-subunits and applied a harmonic potential on the kinesin head to pull the head away from the tubulin, as done previously [21,41]. The pulling force is along the +*y* direction. We took the spring constant as 1000 kJ/mol/nm^2^ and the pulling rate as 0.001 nm/ps, as done previously [21,41]. From the pulling trajectory, snapshots were taken to generate the starting umbrella sampling windows. The simulation period lasted at least for 10 ns to ensure that our results were convergent [21,41]. We took the spacing of the simulation windows to be smaller than 0.2 nm so that the histograms of the configurations overlapped sufficiently with their neighboring windows. The simulation box has a dimension of 14 × 30 × 10 nm^3^, with 14 nm, 30 nm and 10 nm in the *x*, *y* and *z* directions, respectively.

## 5. Conclusions

In this work, with available high-resolution structural data for both kinesin head and unperturbed α/β-tubulin, we studied the effect of the interaction between the kinesin head and α/β-tubulin on the conformation of the α/β-tubulin using all-atom MD simulations. Our simulations showed that the strong interaction of the *ϕ*-head with the α/β-tubulin can rapidly induce (in the order of 10 ns) large conformational changes of the α/β-tubulin, whereas the weak interaction of the ADP-head with the α/β-tubulin induces little conformational changes of the α/β-tubulin. Furthermore, we calculated the binding energy of the head in the strong MT-binding (*ϕ*) state to the α/β-tubulin, the binding energy of the head in the weak MT-binding (ADP) state to the α/β-tubulin with the large conformational changes, and the binding energy of the head in the weak MT-binding (ADP) state to the α/β-tubulin without the large conformational changes. The calculated binding energy of the head in the strong MT-binding (*ϕ*) state to the α/β-tubulin is about 11 *k*_B_*T* larger than that of the head in the weak MT-binding (ADP) state to the α/β-tubulin without the large conformational changes. More interestingly, the calculated binding energy of the head in the weak MT-binding (ADP) state to the α/β-tubulin with the large conformational changes is about 17.3 *k*_B_*T* smaller than (or only about half of) that of the head in the weak MT-binding (ADP) state to the α/β-tubulin without the large conformational changes. Therefore, our results imply that after Pi release from the kinesin head, a very short time period is present before the changed conformation of the local α/β-tubulin returns elastically to the normal unchanged one, when the ADP-head has a very low binding energy to the local α/β-tubulin, which is about 17.3 *k*_B_*T* smaller than (or only about half of) that to other unperturbed α/β-tubulins.

## Figures and Tables

**Table 1:** Definition of systems in the MD simulations.

System	Description	Initial Structural Data: pdb
APO-1	*ϕ*-head bound with initially	head: 4LNU [16]
	unperturbed α/β-tubulin	tubulin: 1JFF [27]
APO-2	*ϕ*-head bound with α/β-tubulin determined from	4LNU [16]
	X-ray crystallography	
ADP-1	ADP-head bound with α/β-tubulin having conformational changes	head: 1BG2 [26] tubulin: simulation for APO-1
ADP-2	ADP-head bound with	head: 1BG2 [26]
	unperturbed α/β-tubulin	tubulin: 1JFF [27]
α/β-tubulin	isolated unperturbed α/β-tubulin	1JFF [27]

**Table 2:** The steady-state average changes in positions of Cα atoms of all residues in β-subunit’s helices H11 and H12.

		Positional Change ± Standard Deviation	
System			
	Δ*X* (nm)	Δ*Y* (nm)	Δ*Z* (nm)
APO-1	−0.24 ± 0.11	0.82 ± 0.14	−1.04 ± 0.20
APO-2	−0.03 ± 0.09	0.28 ± 0.11	0.14 ± 0.13
ADP-1	−0.10 ± 0.07	0.24 ± 0.09	0.23 ± 0.15
ADP-2	−0.08 ± 0.07	0.23 ± 0.09	0.22 ± 0.14
α/β-tubulin	−0.08 ± 0.09	0.38 ± 0.10	−0.22 ± 0.15

## Data Availability

The data that support the findings of this study are available from the corresponding author upon reasonable request.

## Supplementary Materials

The following are available online at https://www.mdpi.com/article/10.3390/ijms22136709/s1.
