# Supplementary material for: Studies of Conformational Changes of Tubulin Induced by Interaction with Kinesin Using Atomistic Molecular Dynamics Simulations
Source: Int J Mol Sci. 2021 Jun 23;22(13):6709. doi: 10.3390/ijms22136709 (PMC8268240; doi:10.3390/ijms22136709)
Supplement: Supplementary file 1 [file ijms-22-06709-s001.zip › ijms-1235164-supplementary.pdf]

Supporting Information

Studies of conformational changes of tubulin induced by interaction with kinesin using atomistic molecular dynamics simulations

Xiao-Xuan Shi [1,2], Peng-Ye Wang [2], Hong Chen [1], and Ping Xie [2,*]

1. *School of Material Science and Engineering, Central South University of Forestry and Technology, Changsha 410004, China*

2. *Key Laboratory of Soft Matter Physics, Institute of Physics, Chinese Academy of Sciences, Beijing 100190, China*

*Corresponding author:

E-mail address: pxie@aphy.iphy.ac.cn

Supporting Figures

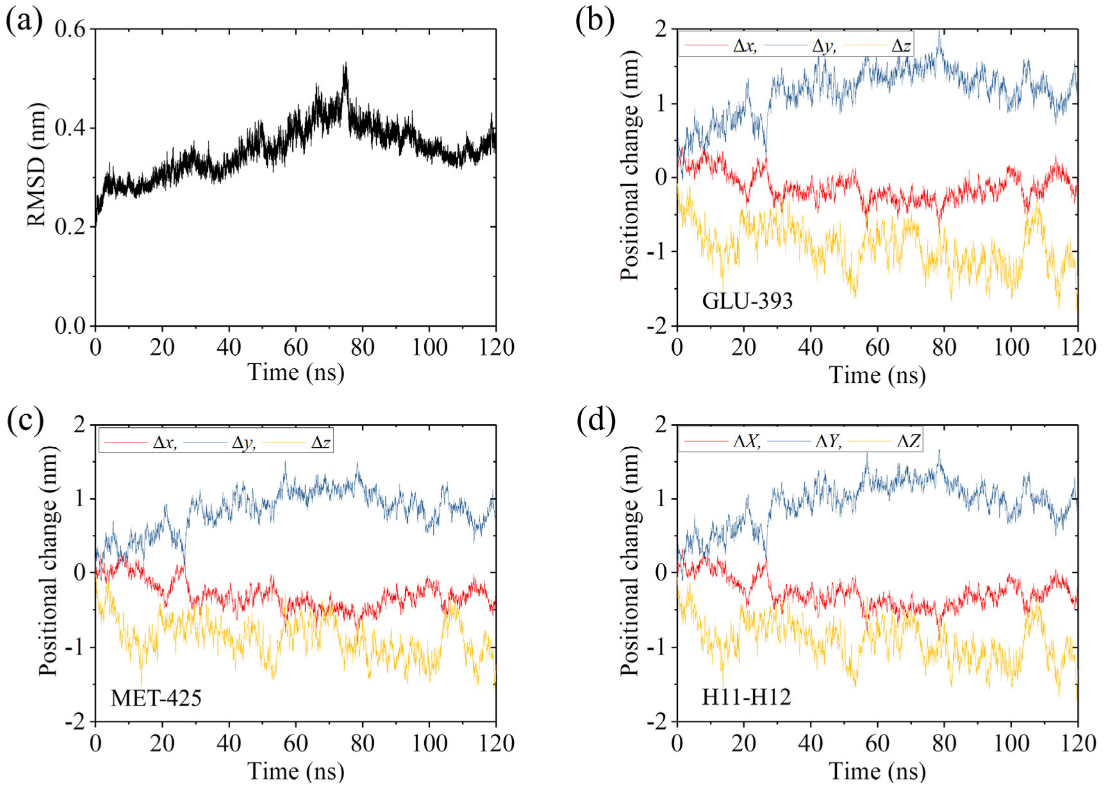

Figure S1. Equilibrium MD simulation results for APO-1 system. **(a)** Temporal evolution of RMSD value. **(b, c)** Temporal evolution of positional changes (Δx, Δy and Δz) of Cα atoms of GLU-393 in H11 and MET-425 in H12 of β-subunit relative to the corresponding ones at t = 0. **(d)** Temporal evolution of the average positional changes (ΔX, ΔY and ΔZ) of Cα atoms of all residues in H11 and H12 of β-subunit.

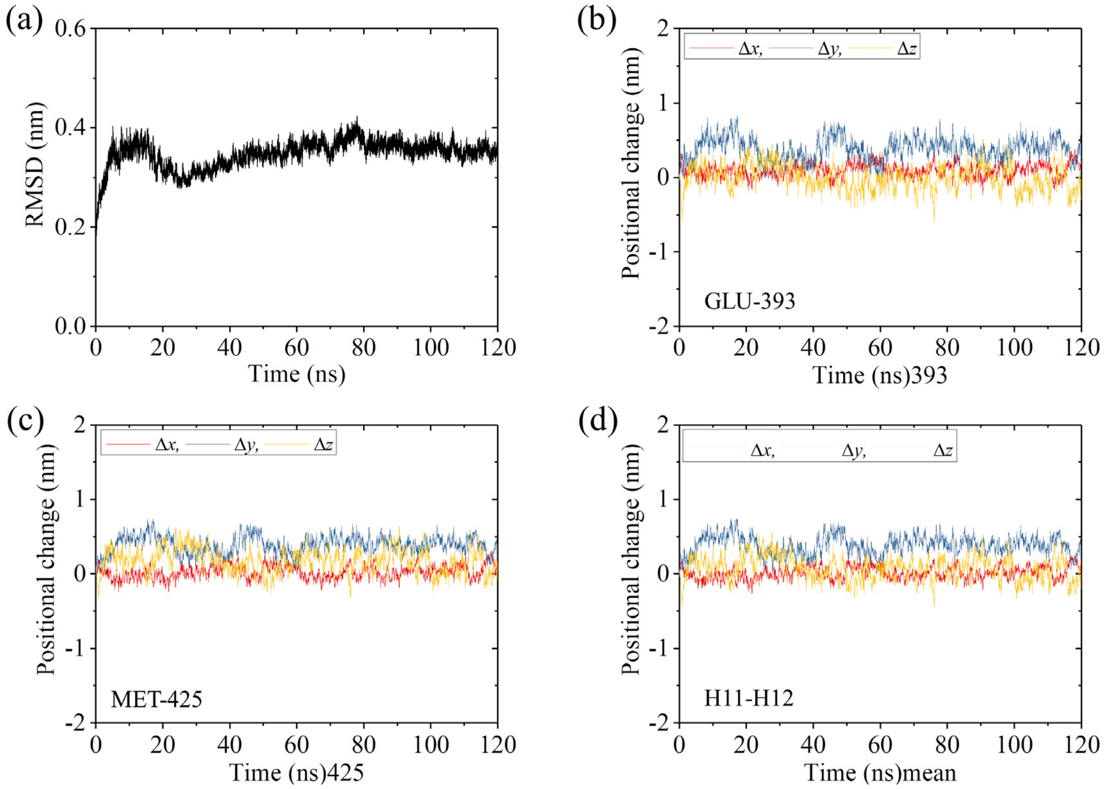

Figure S2. Equilibrium MD simulation results for ADP-2 system. **(a)** Temporal evolution of RMSD value. **(b, c)** Temporal evolution of positional changes (Δx, Δy and Δz) of Cα atoms of GLU-393 in H11 and MET-425 in H12 of β-subunit relative to the corresponding ones at t = 0. **(d)** Temporal evolution of the average positional changes (ΔX, ΔY and ΔZ) of Cα atoms of all residues in H11 and H12 of β-subunit.

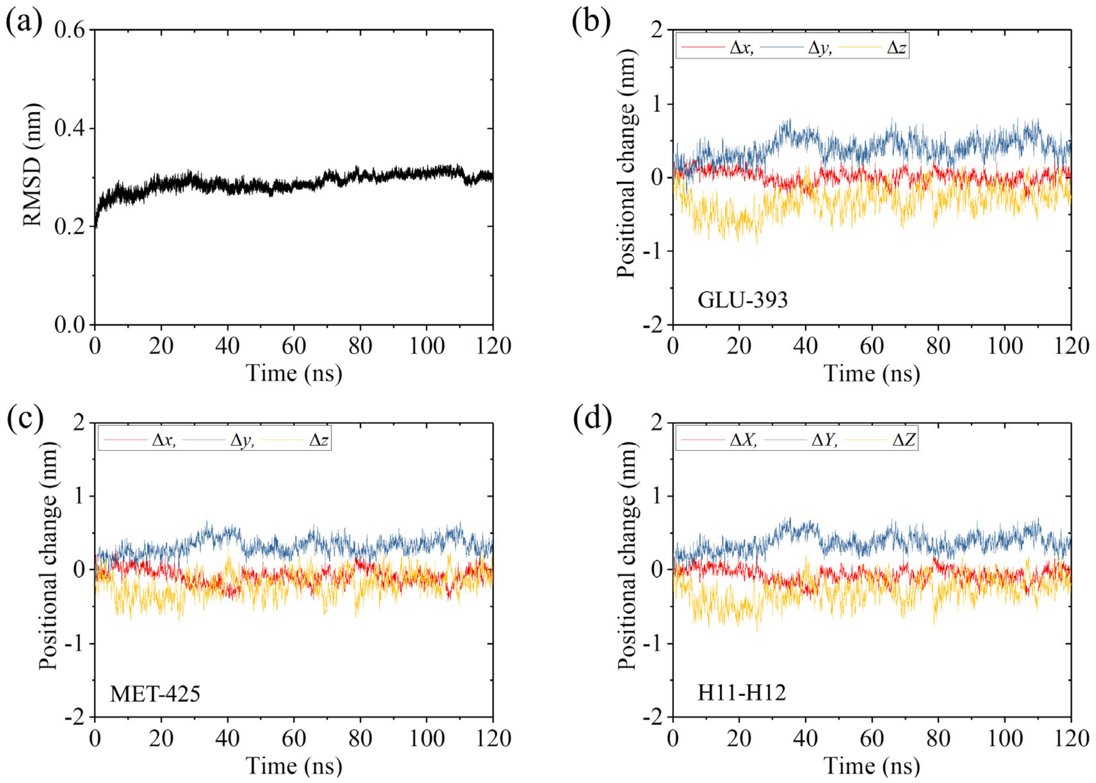

Figure S3. Equilibrium MD simulation results for isolated α/β-tubulin. **(a)** Temporal evolution of RMSD value. **(b, c)** Temporal evolution of positional changes (Δx, Δy and Δz) of Cα atoms of GLU-393 in H11 and MET-425 in H12 of β-subunit relative to the corresponding ones at $t = 0$. **(d)** Temporal evolution of the average positional changes (ΔX, ΔY and ΔZ) of Cα atoms of all residues in H11 and H12 of β-subunit.

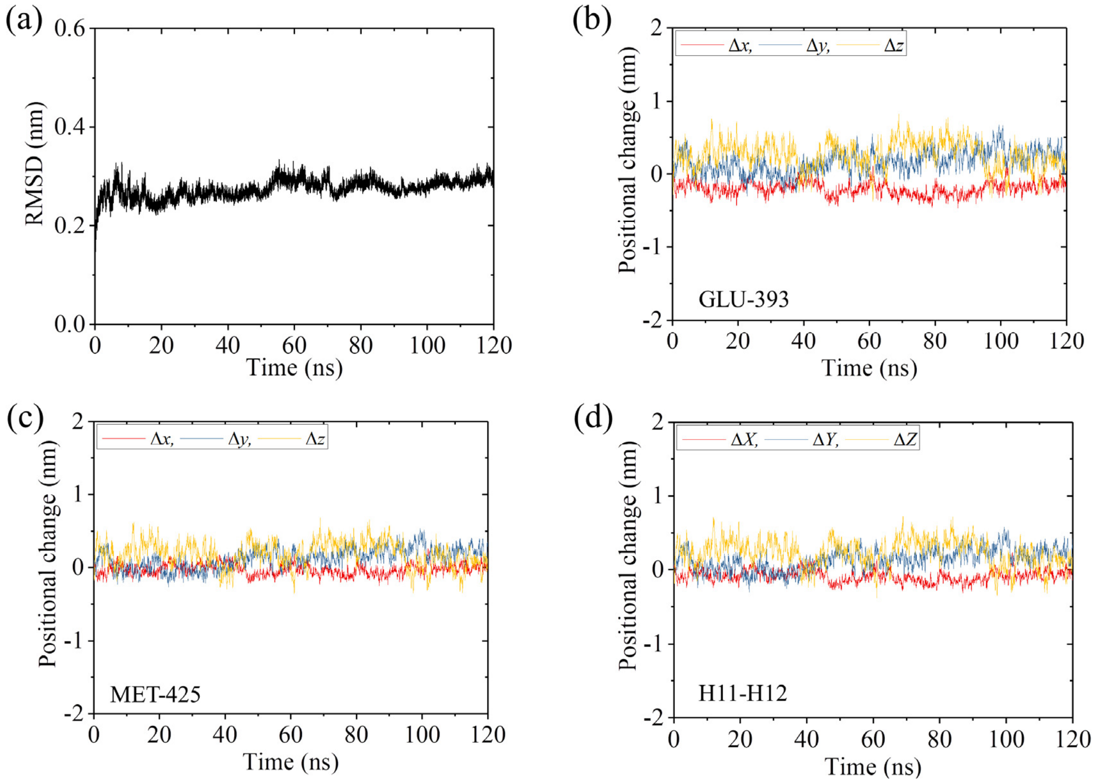

Figure S4. Equilibrium MD simulation results for ADP-1 system. **(a)** Temporal evolution of RMSD value. **(b, c)** Temporal evolution of positional changes (Δx, Δy and Δz) of Cα atoms of GLU-393 in H11 and MET-425 in H12 of β-subunit relative to the corresponding ones at t = 0. **(d)** Temporal evolution of the average positional changes (ΔX, ΔY and ΔZ) of Cα atoms of all residues in H11 and H12 of β-subunit.
